# Phosphorylation-Dependent Regulation of Mammalian Aquaporins

**DOI:** 10.3390/cells8020082

**Published:** 2019-01-23

**Authors:** Veronika Nesverova, Susanna Törnroth-Horsefield

**Affiliations:** Department of Biochemistry and Structural Biology, Center for Molecular Protein Science, Lund University, Box 124, 221 00 Lund, Sweden; veronika.nesverova@biochemistry.lu.se

**Keywords:** aquaporin, protein phosphorylation, membrane channel, regulation by phosphorylation, gating, trafficking

## Abstract

Water homeostasis is fundamental for cell survival. Transport of water across cellular membranes is governed by aquaporins—tetrameric integral membrane channels that are highly conserved throughout the prokaryotic and eukaryotic kingdoms. In eukaryotes, specific regulation of these channels is required and is most commonly carried out by shuttling the protein between cellular compartments (trafficking) or by opening and closing the channel (gating). Structural and functional studies have revealed phosphorylation as a ubiquitous mechanism in aquaporin regulation by both regulatory processes. In this review we summarize what is currently known about the phosphorylation-dependent regulation of mammalian aquaporins. Focusing on the water-specific aquaporins (AQP0–AQP5), we discuss how gating and trafficking are controlled by phosphorylation and how phosphorylation affects the binding of aquaporins to regulatory proteins, thereby highlighting structural details and dissecting the contribution of individual phosphorylated residues when possible. Our aim is to provide an overview of the mechanisms behind how aquaporin phosphorylation controls cellular water balance and to identify key areas where further studies are needed.

## 1. Introduction

Phosphorylation is the most studied post-translational protein modification, allowing for simple and reversible regulation of protein function. It is estimated that around 30% of human proteins are phosphorylated at some point in their lifetime [1]. Protein phosphorylation can lead to a myriad of effects, for example altered biological activity, induction of structural changes, marking protein for cellular translocation and preventing or initiating protein-protein interactions [1]. The phosphorylation process is governed by a tight interplay of kinases and phosphatases, and is often tissue-specific, thereby allowing proteins to be regulated based on the need of a particular tissue [2].

For the ubiquitous membrane-bound water channels known as aquaporins (AQPs), phosphorylation is an important regulatory mechanism. In eukaryotes, this allows water flow across cellular membranes to be tightly regulated in response to various external and internal signals, thereby maintaining the desired water balance of the organism [3]. Fast regulation can be achieved by two means—either by altering the water permeability rate through the pore itself (gating) or by rapidly changing the abundance of AQP molecules in the plasma membrane by shuttling the protein between the plasma membrane and intracellular vesicles, so-called trafficking [4]. Traditionally, AQPs are divided into two main sub-groups: Orthodox AQPs, that transport water only, and aquaglyceroporins, which in addition to water also facilitate the transport of other small uncharged solutes, such as glycerol and urea [5]. Additionally, a third sub-group, the intracellularly located superaquaporins, has been identified in recent years, for which the substrate specificity and cellular roles remain to be conclusively determined [6].

**Figure 1:**
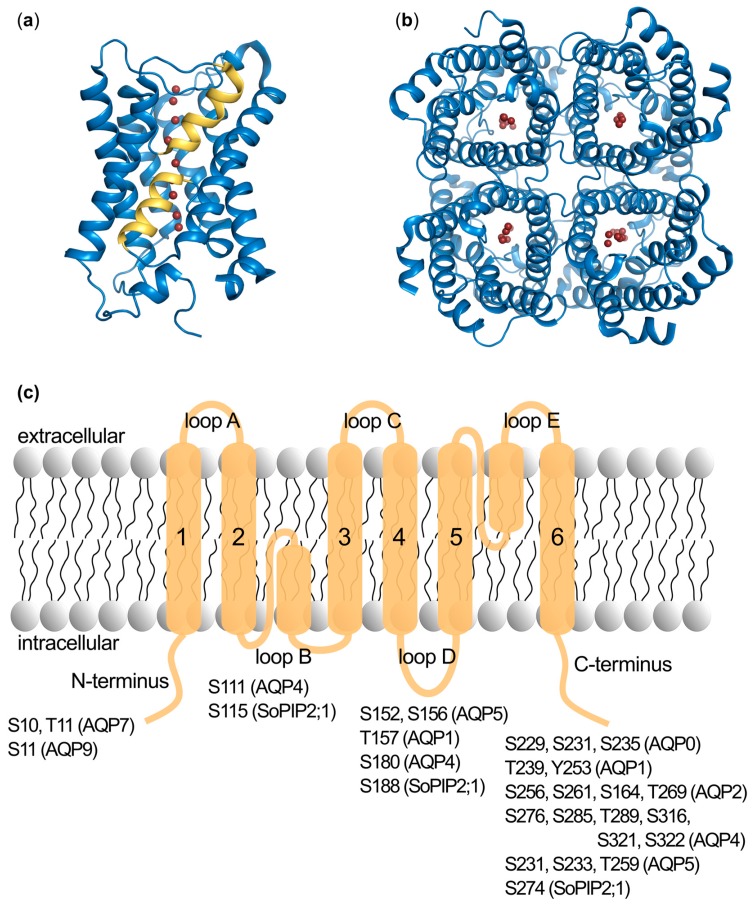
The overall structure of aquaporins and the localization of individual phosphorylation sites. (**a**) Side view of the aquaporin monomer represented by the crystal structure of human AQP5 (PDB 3D9S). Water molecules in the water-conducting pore are marked as red spheres. The two half-helices formed by loops B and E are coloured yellow. (**b**) The tetrameric assembly from the cytoplasmic side. (**c**) A schematic representation of the aquaporin topology with all phosphorylation sites identified in mammalian aquaporins as well as the spinach aquaporin SoPIP2;1 marked.

All AQPs share the same structural features with four monomers assembling as a tetramer (Figure 1). Each monomer comprises six alpha-helices that span the membrane and form a narrow pore together with two half membrane-spanning helices which contain the conserved asparagine-proline-alanine (NPA) motifs. Within this pore, water passes at almost the diffusion rate along an osmotic gradient. Phosphorylation sites involved in regulation are commonly found in the N- and the C-termini, which are located in the cytoplasm as well as in the cytoplasmic loops B and D (Figure 1c).

Thirteen AQPs (AQP0-12) have been identified in mammals, which are expressed and regulated in a tissue-dependent manner [7,8]. A majority of these have been shown to be post-translationally regulated, either through altered water transport through individual AQPs or, more commonly, by changing the number of AQP molecules present in the plasma membrane through trafficking [9]. A common denominator for both regulatory processes is that they are governed by phosphorylation, primarily by altering interactions with regulatory proteins or the cytoskeleton and microtubule system [10]. The aim of this review is to summarize what is currently known about phosphorylation-dependent regulation of mammalian AQPs, specifically focusing on those for which phosphorylation has been most extensively studied. In addition, we will give a brief overview of the most common methods for studying phosphorylation at specific sites and its effect on AQP function in cells as well as in the test tube. We hope that this will help identify knowledge gaps and inspire future efforts in the field.

## 2. Methods Used to Study Phosphorylation Sites in Aquaporins

Phosphorylation of AQPs can be readily identified in cells, tissues, as well as cell lysates and purified proteins using phosphospecific antibodies [11]. However, to fully understand phosphorylation-dependent AQP regulation, the coupling between phosphorylation at specific sites and AQP structure, function and sub-cellular localization must be determined. There are several methods that can aid in these types of studies, a selection of which is summarized below.

### 2.1. Identification of Phosphorylation Sites

The first step, identification of phosphorylation sites is most often performed using various prediction servers, like the NetPhos Server [12], where the amino acid sequence of the protein is scanned for the presence of consensus kinase phosphorylation sites. While this identifies residues with the potential to be phosphorylated, it does not give any evidence for an actual phosphorylation in vivo, although the accessibility of the site may be an indication. Such information can instead be obtained through phosphoproteomics and the use of mass spectrometry, which can reliably assign phosphorylation to a residue from native samples. In these methods, antibodies or TiO_2_ are used to facilitate the specific enrichment of phosphopeptides. In rats, phosphoproteomics has been studied extensively and the results can be found in the CPR PTm Resource, an online database of more than 30,000 identified protein phosphorylation sites, including those in aquaporins, across 14 different rat tissues [13]. In vitro phosphorylation can also give an initial hint on whether a protein or a peptide could be a substrate for a particular protein kinase. This is often done using ^32^P-labelled ATP and auto-radiographic detection on an SDS-PAGE gel as used, for example, to study the phosphorylation of AQP1 by PKC [14], or by the use of phosphorylated kinase substrate-specific antibodies, as in the case of phosphorylation of AQP7 by PKA [15].

### 2.2. Phosphorylation Site Mutations

Once phosphorylation sites have been identified the effect of these on AQP structure and function can be studied. To pinpoint the effect of specific phosphorylation sites, point mutations are most often introduced, replacing the phosphorylated residue with a phospho-mimicking counterpart. For phosphorylated serine or threonine this is commonly an aspartic or glutamic acid, whereas a phosphorylated tyrosine can be mimicked by the non-natural amino acid analogue p-carboxymethyl-l-phenylalanine (pCMF) [16]. For AQPs, a wide range of studies using phospho-mimicking mutants in cells and in vitro have helped determine the effect of phosphorylation on AQP structure [17,18,19], protein-protein interactions [20,21,22], sub-cellular localization [19,23,24,25] and channel permeability [14,26,27,28].

In cell-based studies, mutants designed to abolish phosphorylation, typically by replacing the phosphorylated residue with alanine (or phenylalanine in the case of tyrosine), are also created. Since these residues are unable to be phosphorylated in the cell, this ensures a phosphorylation-free background, enabling the comparison of non-phosphorylated protein with the phospho-mimicking mutants. In studies of purified proteins, alanine/phenylalanine mutants are not necessary, since the protein can be in vitro dephosphorylated by the addition of a phosphatase. Recombinantly expressed proteins that are meant for purification may become phosphorylated during expression, as has been shown for some AQPs [21,29]. It is therefore important to check the phosphorylation status of the purified protein before using it for further studies, and dephosphorylate, if necessary.

### 2.3. Functional Effects of Phosphorylation at Specific Sites

The effect of phosphorylation on AQP permeability is typically characterized using a shrinking/swelling assay where AQP-expressing cells or AQP-containing proteoliposomes are subjected to an osmotic shock and the change in cell or liposome volume is followed by measuring light scattering intensity. Proteoliposomes allow the most direct comparison of phospho-mimicking mutants and wild-type AQP, since the proteins are purified prior to reconstitution. This setup was, for example, used to study the effect of phosphorylation on the water permeability of AQP2 [30]. Cell-based systems have the advantage of enabling the use of kinase/phosphatase effectors and other trigger molecules, in addition to the effect of point mutations, and also avoid the need for protein purification. However, due to the complexity of the system, the results can be difficult to interpret, since effectors and trigger molecules affect a wide range of proteins and cellular processes, and the AQP of interest may, or may not, reach the plasma membrane. *Xenopus* oocytes are commonly used for cell-based AQP permeability assays and have, for example, been used to study the effect of phosphorylation on AQP1 [14] and AQP4 [31]. Mammalian cell lines are also used, allowing a particular AQP isoform to be studied in its native cell type, for example AQP4 in astrocytes [32] or AQP2 in collecting duct cells [22]. While using primary mammalian cells and the study of intrinsically expressed AQPs provide the most native environment, this method cannot be used to study the effect of abolishing or mimicking phosphorylation by point mutations.

AQP phosphorylation also affects membrane water permeability by altering AQP sub-cellular localization. One of the most commonly used methods to characterize this is fluorescent confocal microscopy. In these studies, AQP spatial localization in response to point mutations or cellular effectors is determined using cells that are expressing fluorescently tagged AQP [23,33,34], or by using immunolabelling methods [35,36]. The presence of a protein in the plasma membrane can also be studied using cell surface biotinylation, a method that is able to distinguish and quantify protein localization on the apical versus basolateral membrane, as was done to study the trafficking polarization of AQP4 [37]. As for cell-based water permeability methods, care has to be taken when evaluating the results in order to elucidate what effects are directly related to AQP phosphorylation. Furthermore, the fluorescent tag itself may influence the sub-cellular localization of the AQP, as has been shown for AQP5 [34], and therefore also needs to be taken into account.

## 3. Kinases and Phosphatases in Human AQP Regulation

Protein kinases are key regulatory enzymes, reversibly attaching a phosphate group onto serine, threonine and tyrosine residues. The human kinome contains more than 500 kinases, which mediate most of the signal transduction [38]. Commonly, the kinases need to be phosphorylated themselves in order to become active. As can be seen in Table 1, the majority of AQP0-AQP9 are phosphorylated by a member of the AGC kinase subfamily, which contains some of the most well described kinases like protein kinase A (PKA), protein kinase C (PKC), and protein kinase G (PKG). AGC kinases share a conserved structure, where the catalytic domain comprises two lobes that sandwich one ATP molecule, which serves as a phosphate donor. Within the AGC subfamily, the enzymatic activity is not very specific and many kinases were found to phosphorylate the same substrate. Hence, the sub-cellular localization of the kinases is the key factor that decides what substrates will be phosphorylated by which kinase [39], allowing one pathway to be regulated by multiple stimuli. Typically, the AGC kinase subfamily substrates contain the basic residues arginine or lysine upstream of the residue to be phosphorylated. The consensus for PKA is Arg–Arg–X–Ser/Thr or Arg-X-Ser/Thr, for PKC it is Arg–Lys–X–Ser/Thr–X–Arg/Lys and for PKG Arg/Lys_2–3_–X–Ser/Thr [39].

Both PKA and PKG are cyclic nucleotide-dependent protein kinases that are regulated by binding the second messengers cAMP and cGMP, respectively [39]. It has been shown that, upon binding of cyclic nucleotides, PKA and PKG undergo structural rearrangements, that are necessary for revealing the substrate binding site. Conventional PKC isoforms are dependent on binding of calcium which causes a structural rearrangement and final activation of the enzyme [39]. There are, however, also atypical PKC isoforms, which do not require calcium, one of them being PKC_ζ_ that is involved in phosphorylation of AQP9 [40].

For AQP4, two additional kinases that do not belong to the AGC subfamily have been identified to modulate protein function [37,41]: Calmodulin-kinase II (CaMKII), which is activated by the binding of calmodulin upon the intracellular Ca^2+^ signal, and casein kinase II (CKII), which seems to be constitutively activated and regulated by various pathways. It is probable that other kinases are also involved in the aquaporin regulation. Further serial phosphoproteomic analyses of aquaporins in native tissues, with various kinases inhibited, would shine further light on the physiological pathways of aquaporin phosphorylation.

An important property of phosphorylation as a regulatory mechanism is the ability to be fully reversible through the dephosphorylating activity of phosphatases. Although this is as equally important for protein regulation as the phosphorylation process itself, the knowledge of how aquaporin dephosphorylation is regulated is very limited. The role of specific phosphatases have been most studied for AQP2, indicating the involvement of several phosphatases, including PP1, PP2A, PPA2B and PP2C (calcineurin) [60,61,62], although many of the details remain to be elucidated. As such, this area of aquaporin regulation represents a vast knowledge gap, which should be addressed, in particular since protein phosphatases are attractive drug targets, an example being the calcineurin inhibitors, which are already on the market and used as immuno-suppressives [63].

## 4. Modulation of Protein-Protein Interactions upon Phosphorylation

In mammalian cells it is not necessarily the phosphorylation itself that causes aquaporin gating or trafficking. Instead, it functions as a signal, modulating the affinity towards a regulatory protein, that either directly affects the water transport or ensures effective exo- or endo-cytosis, thereby targeting the protein to a specific sub-cellular localization. From a structural point of view, the addition of a phosphate group onto a residue causes an increase in bulk, as well as adds a negative charge. This can directly impair binding if the phosphorylation site is located within the interaction surface, a mechanism that is thought to regulate the binding of calmodulin to AQP0 [64]. Phosphorylation also impairs the binding of perilipin 1 to AQP7, thereby allowing AQP7 to reach the adipocyte plasma membrane, increasing its glycerol permeability [15]. The perilipin binding site is, however, not known wherefore it is difficult to predict the mechanism by which phosphorylation regulates the affinity in this case. The addition of a phosphate group could also help create a binding site, thereby increasing the affinity to a regulatory protein. Phosphorylation of S274 of AQP4 has this effect—it increases the affinity of AQP4 towards the μ-subunit of the AP2 adaptor complex [37]. This adaptor complex then binds clathrin, a protein which initiates vesicle formation during endocytosis and plays an essential role in intracellular trafficking of many membrane proteins [65].

Phosphorylation of residues outside the regulatory protein binding site could still potentially regulate the complex formation. This has been shown for AQP2, in which case phosphorylation of the AQP2 C-terminus allosterically decreases its affinity towards lysosomal trafficking regulator protein 5 (LIP5), thereby targeting AQP2 to multi-vesicular bodies and lysosomal degradation. It was further shown that this was controlled in a phosphorylation-site specific manner with the four different known phosphorylation sites exhibiting different effect on LIP5 affinity [21].

To date, many interaction partners, other than those mentioned above, have been identified for aquaporins, especially for AQP0, AQP2 and AQP4 [66], and we can expect that many more will be identified in the future. The effect of protein phosphorylation on these interactions is to the most extent unknown, but is likely to play a prominent role. However, not every interaction is governed by phosphorylation, as shown in the study, where the binding of filensin to AQP0 seems to be completely phosphorylation-independent [67].

## 5. Phosphorylation-Dependent Aquaporin Gating

**Figure 2:**
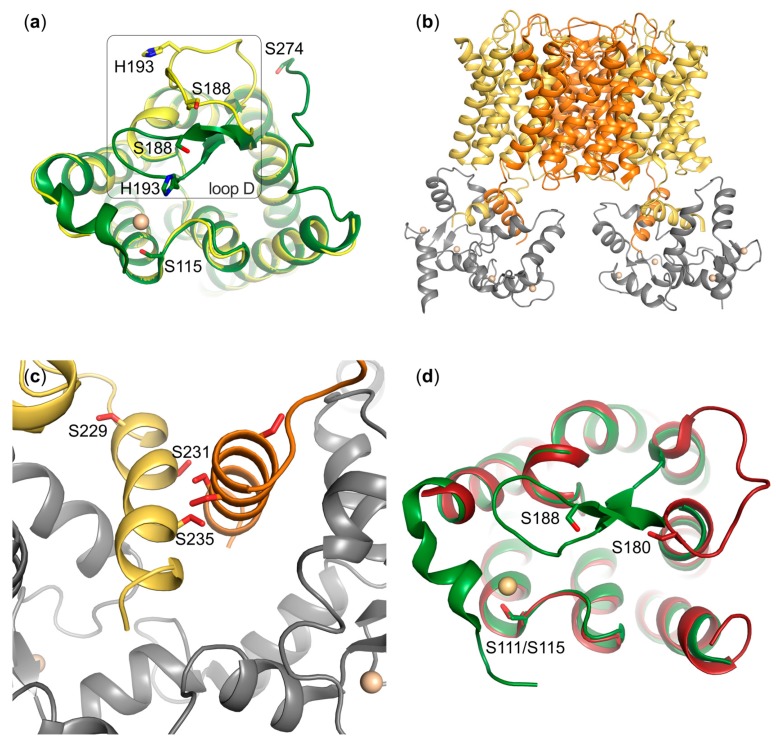
Structural features of aquaporin gating. (**a**) Comparison of the SoPIP2;1 crystal structures in closed (green, PDB code 1Z98) and open (yellow, PDB code 2B5F) conformations. Residues involved in gating are highlighted in stick representation. In the closed conformation, loop D is held in place by an interaction with a Cd^2+^-ion (beige), representing an in vivo Ca^2+^-site, thereby occluding the channel. Phosphorylation of S115 as well as S274 from the neighbouring monomer destabilize the closed state. (**b**) Model of the complex between AQP0 (yellow and orange) and calmodulin (grey) based on a 25Å pseudoatomic cryo-EM structure (PDB code 3J41). Two molecules of calmodulin bind to one AQP0 tetramer and this interaction results in closure of the water pore. (**c**) Zoom-in on the interaction site in the AQP0-calmodulin complex, showing how each molecule of calmodulin binds two short C-terminal helices from neighbouring AQP0 monomers. Identified phosphorylation sites in AQP0 are highlighted. The phosphorylation of these residues affects calmodulin binding and water permeability of AQP0. (**d**) Crystal structure of human AQP4 (red, PDB code 3GD8) overlaid with the closed structure of SoPIP2;1 (green). Phosphorylation sites in loop B (S111/S115) and loop D (S188 and S180) are highlighted. Loop D in AQP4 is significantly shorter than in SoPIP2;1.

In situ regulation of aquaporins in the membrane through direct gating is a common regulatory mechanism in plants, allowing them to cope with rapid changes in water supply. For plant aquaporins located in the plasma membrane (plasma membrane intrinsic proteins, PIPs), phosphorylation of two highly conserved serine residues during drought induces channel opening, whereas low pH and high Ca^2+^ trigger channel closure during periods of flooding [68]. Based on the X-ray structures of both the closed and the open conformation of the spinach aquaporin SoPIP2;1 [69], a gating model unifying these three events has been proposed. The structures showed that gating is achieved through conformational changes of the cytoplasmic loop D and that the closed state is stabilized through interactions with a divalent cation binding site at the N-terminus, harbouring a Cd^2+^-ion in the structure, but binding Ca^2+^ in vivo (Figure 2a). The phosphorylation of S115 in loop B, as well as S274 in the C-terminus, destabilize the closed state thereby allowing the channel to open. An additional phosphorylation site is predicted at S188 and has been shown to increase water permeability in vitro, however the importance of this site remains to be established in vivo [17]. In contrast, protonation of a conserved histidine (His193) on loop D stabilizes the closed conformation by forming hydrogen bonds with residues in the Ca^2+^-binding site or loop B [70].

In mammals, gating has been shown to regulate AQP0 in the eye lens through phosphorylation-dependent binding of the regulatory protein calmodulin (CaM) [64], which has also been suggested to be gated by pH as well as junction-formation [71,72]. Moreover, the recent crystal structure the human aquaglyceroporin AQP10 suggests that a pH-dependent gating mechanism controls glycerol flux, which was supported by functional studies and molecular dynamics simulation [73]. The gating of other mammalian AQPs has been suggested, in particular for AQP4 [32], but remains to be conclusively established. For AQP1, there is some evidence that the central pore created by the tetrameric arrangement of AQP monomers functions as a cGMP-gated cation channel [14,74,75,76,77], however this is also a matter of controversy. Below we review what is currently known about the role of phosphorylation in AQP0 gating, as well as the literature on phosphorylation-dependent gating of AQP4 and AQP1.

### 5.1. AQP0

AQP0 represents the most abundant membrane protein of the human eye lens, where it plays a key role in upholding the lens water microcirculation system. In addition, it functions as an adhesion protein, whereby AQP0 tetramers in adjacent fibre cells in the lens core interact with each other, forming tight junctions that are important for lens transparency [78]. The water transport through AQP0 is controlled by the interaction with CaM, a key soluble regulatory protein [71,79], which binds to the hydrophobic side of a short amphipathic helix located in the AQP0 C-terminus (residues 225–263) (Figure 2b) [80]. The first structural model of the AQP0-CaM complex proposed a non-canonical binding, where CaM in its Ca^2+^-bound form interacts with two C-terminal helices belonging to neighbouring AQP0 monomers [81], and it was recently shown that this binding is a highly cooperative process [82]. A 25Å pseudo-atomic single particle cryo-EM structure of the cross-linked complex, confirmed the complex stoichiometry, and it was suggested that CaM-binding allosterically inhibits water transport by inducing conformational changes of the residues in the water-conducting pore [64].

Several studies have shown that AQP0 phosphorylation plays a role in CaM-dependent gating. The AQP0 C-terminus contains several phosphorylation sites: S229 (consensus for casein kinase II) [43], S231 (consensus for PKC) [43] and S235 (consensus for PKA) [83] (Figure 1c and Table 1), as determined by mass spectrometry). PKA was found to phosphorylate human S235, and this was mediated by a direct interaction of A-kinase anchoring protein 2 (AKAP2) with AQP0. AKAP2 was found to bind to residues 238–246 of a peptide corresponding to AQP0 C-terminus, and upon this binding it could anchor PKA for efficient phosphorylation of S235 [42]. Binding of CaM to the C-terminal helix of AQP0 is severely impaired when either S231, S235 or both are phosphorylated as tested with AQP0 C-terminal peptides [84]. In oocytes, water permeability increased for S229D and S231D mutants, and the regulation of permeability by extracellular calcium was lost. The opposite effect was shown for the S235D mutant, which exhibited decreased permeability in the presence of calcium. The fact that the S235D mutant retained its calcium sensitivity, suggests that it might still be able to bind CaM. The double mutant S229D/S235D also showed a decreased permeability that was not affected by Ca^2+^ [20]. PKC-dependent phosphorylation of S235 has also been shown to be involved in AQP0 trafficking and is a prerequisite for efficient AQP0 translocation to the plasma membrane after synthesis [44].

Although several functional studies have probed the effect of various modifications of the C-terminus, including phosphorylation, on the water permeability of AQP0, very little is known about how these modifications structurally affect the binding of CaM. S231 and S235 are located within the CaM-binding domain, albeit on the hydrophilic side of the amphipathic C-terminal helix (Figure 2c). Nevertheless, in the low-resolution structural model of the AQP0-CaM complex they are still proposed to participate in the interaction and it is therefore easy to imagine that phosphorylation would impair this binding. Recently, a Brownian Dynamics (BD) docking simulations suggested loop D as a second site of contact between AQP0 and CaM. In this model, CaM binds to the C-terminal helix but also forms electrostatic interactions with the arginines of loop D, thereby further stabilizing the complex and providing a mechanical coupling of CaM to the pore gating residues [85]. This model is further supported by the previous functional data on S235 phosphorylation: The phosphorylated C-terminal peptide was unable to bind CaM in vitro [81], while the phosphorylated full-length AQP0 retained some calcium sensitivity in oocytes [20]. This suggests that the additional interaction interface might play a role in defining the strength of the complex. The presence of a second interaction site could also explain why S229D mutation increases the water permeability in calcium-insensitive manner, in vivo [20]. In the BD simulation of the complex, phosphorylation of S229 did not impair CaM binding per se, but it shifted the way CaM bound to the D loop in a way that the binding no longer caused the allosteric closure of the pore [85]. In conclusion, these data suggest that the previous binary model of the pore as either closed or open, in the CaM-bound and unbound states respectively, might in reality be much more complex with an intricate phosphorylation-dependent regulation.

### 5.2. AQP4

AQP4 is found in several tissues in the body, but has received the most attention for being the predominant aquaporin in the brain and the central nervous system [86]. It is highly expressed in areas which are in contact with cerebrospinal fluid and is particularly abundant in astrocytic end-feet surrounding blood vessels [87]. This suggests that AQP4 plays an important role in fluid exchange within the brain and studies show that it may be exploited for treatment of brain edema [88,89].

**Figure 3:**
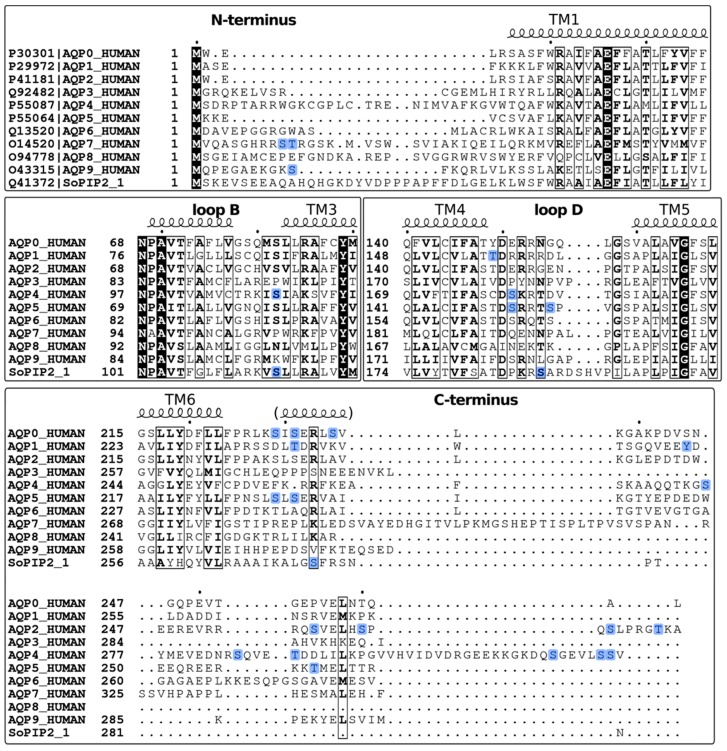
Sequence alignment of human AQP0-AQP9 and spinach aquaporin SoPIP2;1. The corresponding UniProt IDs are marked in the top panel. Secondary structure information was based on sheep AQP0 (PDB code 2B6O). The alignment was generated using PSI/TM-Coffee server [92] and visualized using ESPript 3.0 [93]. Residues that are strictly conserved are highlighted with black background and similar residues are highlighted in boxes. Phosphorylation sites are highlighted in blue.

AQP4 has been proposed to be gated in a phosphorylation-dependent manner, however this is still a subject of controversy. When rat astrocytes expressing AQP4 were stimulated with glutamate, the water permeability increased without any change in the AQP4 cellular localization, suggesting that glutamate might regulate gating [32]. This effect was attributed to phosphorylation of S111 in the cytoplasmic loop B [90]. Interestingly, S111 corresponds to S115 in spinach aquaporin SoPIP2;1 (Figure 2d and Figure 3) which, when phosphorylated, helps maintain the pore in an open conformation by disrupting the interaction between loop D and the N-terminus (see above) [68]. It was therefore suggested that, similarly to SoPIP2;1, AQP4 gating might also be regulated by phosphorylation of this residue [26]. However, this is not supported by the crystal structure of human AQP4, which shows the channel in an open conformation in the absence of phosphorylation. Moreover, the loop D is considerably shorter in AQP4 than in SoPIP2;1 and it is difficult to see how it would reach to block the channel [91]. If AQP4 is indeed gated, it is more likely to employ another mechanism to occlude the channel, for example by using one of the termini for which there is currently no structural information, possibly by interacting with S111.

Several functional studies have tried to shine light on the matter and to identify the kinase involved in phosphorylation of S111, however a consensus has so far not been reached. In rat astrocytes expressing AQP4, the water permeability increase, induced by extracellular potassium levels or by lead, was abolished by inhibition of PKA [53] or CaMKII [41] respectively. Similarly, in S111A mutant-expressing rat astrocytes, the stimulation by glutamate or lead was abolished [32,41]. In vitro phosphorylation on AQP4 peptides corresponding to loop B confirmed that S111 might be a substrate for PKG, but not for CaMKII. This suggests that CaMKII does not directly phosphorylate AQP4, but instead triggers phosphorylation of nitric oxide synthase (NOS) and thereby activates the NO-cGMP-PKG signalling pathway. This theory was further strengthened by experiments using nitric oxide donors and nitric oxide synthase inhibitors [32]. In contrast, experiments in oocytes show no observable effect on the cell swelling when mutating S111 to alanine or to glutamic acid. Also, neither activators nor inhibitors of PKA and PKG had any effect on the water permeability of AQP4 in oocytes, and PKG activators did not had any effect on rat primary astrocytes. In the same study phosphorylation of S111 could not be detected in vivo [94].

Another residue that has been suggested to play a role in AQP4 gating mechanism is S180, which is a consensus PKC site located in loop D (Figure 1c and Figure 2d). In LLC-PK1 cells expressing N-terminally GFP-tagged mouse AQP4, PKC activators caused a decrease in water permeability that was not observed in un-transfected cells and that could be reversed by PKC inhibitors. Mutating S180 to alanine abolished this effect, leading the authors to conclude that phosphorylation of this residue is likely to be responsible for the PKC-induced effects. Since confocal microscopy did not reveal any effect on AQP4 sub-cellular localization, it was proposed that AQP4 was gated in a PKC-dependent manner [26]. This is, however, contradicted by studies in oocytes, where non-tagged AQP4 was internalized upon treatment with vasopressin, and this was regulated by PKC. The S180A mutation decreased this internalization in oocytes that had been treated with PKC-activators, implying a role of S180 in trafficking and not gating [27]. An electron crystallography structure of the S180D mutant shows that this mutation does not induce any conformational change, as the structure was virtually identical to the structure of wildtype AQP4 [18]. As discussed above for S111, however, it is possible that S180 in its phosphorylated state could interact with the AQP4-termini, thereby occluding the channel. Taken together, more studies are needed, in particular structural characterization of full-length AQP4, before we can confirm or rule out gating as a regulatory mechanism.

### 5.3. AQP1

AQP1 is found in a wide range of tissues, including kidney, eye, lung, red blood cells, muscle, and brain tissue [7,95]. Apart from its obvious function as a water channel in these tissues, several studies have suggested AQP1 to also act as a non-selective monovalent cation channel [14,74,75,76,77], although controversies exist depending on the expression system used [76,96]. In contrast to water transport, where each monomer functions as an individual water channel, ions are believed to be conducted through the central channel, created by tetrameric assembly. Studies in oocytes showed that the transport of ions through AQP1 was activated by cGMP, and that cGMP was shown to interact with AQP1-containing membranes. It was, therefore, suggested that AQP1 functions as an cGMP-gated ion channel [75]. Through molecular dynamics simulations, a possible mechanism for this could be deduced, whereby cGMP binds to arginine residues at the cytoplasmic face of AQP1, which induced conformational changes in loop D that allowed the central channel to open [97]. A C-terminal phosphorylation site was identified at Y253 through immuno-precipitation analysis and it was shown that mutating this residue to cysteine disrupted the cGMP-activated ion current [46].

## 6. Phosphorylation in Human Aquaporin Trafficking

The majority of human AQPs have been proposed to be regulated by trafficking [3]. For some AQPs, this involves constitutive targeting to the plasma membrane after synthesis, while others are kept in storage vesicles, waiting for an appropriate trigger before translocation occurs, so-called regulated exocytosis [98]. The effective and rapid removal of the protein from the plasma membrane via endocytosis is of equal importance in controlling its plasma membrane abundance, after which the AQP can be stored in cytoplasmic storage vesicles for re-use or degraded in the lysosome. A number of intracellular and extracellular translocation triggers has been identified for AQPs, including hormones, signalling molecules, and changes in tonicity (see [3] for a review). The diversity of these triggers suggests that a multitude of different pathways is involved. The use of multiple pathways is particularly important when considering that certain cells may contain several AQP isoforms that need to be regulated at least partly independently. Nevertheless, there are some features that remain similar, such as the involvement in microtubule-dependent vesicle trafficking system as well as reversible phosphorylation by kinases/phosphatases [3]. For human AQPs, phosphorylation-dependent trafficking is best characterized for AQP1, AQP2, AQP4, and AQP5, a summary of which is given below.

### 6.1. AQP1

Trafficking of AQP1 has been proposed to involve both PKA- and PKC-dependent pathways, of which the PKC-pathway is best characterized. In oocytes, treatment with a PKC activator resulted in higher levels of AQP1 in the plasma membrane [14]. PKC is also involved in rapid translocation of AQP1 to the plasma membrane in response to a hypotonic stimulus, as shown in both HEK293 cells and astrocytes [24,99]. In HEK293-cells, this was further demonstrated to be mediated by Ca^2+^ influx through TRPC1 channels, resulting in activation of PKC and calmodulin and subsequent phosphorylation of AQP1 [24]. Contradicting results have been obtained from human brain tumor cells stably transfected with AQP1, which were unaffected by changes in PKC activity [28]. The discrepancies between these results may be due to tissue-specific effects and/or the involvement of different PKC isoforms.

Two phosphorylation sites are implied in PKC-mediated AQP1 regulation: T157, located in loop D, and T239 of the C-terminus (Figure 1c and Table 1), as first suggested by in vitro PKC phosphorylation of corresponding peptides [14]. The specific role of these phosphorylation sites in hypotonicity-induced translocation was probed by mutational studies in which one or both threonines were replaced by alanine or aspartate. T157A, T239A, T157D, and T239D mutants were all able to translocate to the plasma membrane in a hypotonic medium in live imaged HEK293 cells. However, the double mutant T157A/T239A did not translocate upon hypotonic stimulus, showing that phosphorylation of at least one of these sites is necessary for PKC regulated trafficking in response to hypotonicity [24]. In an analogous manner, PKC-dependent AQP1 trafficking has also been studied in terms of regulating the proposed ion-conducting activity of AQP1 (see gating section above) [14]. In the T157A/T239A mutant, the cation permeability regulation by PKC was almost completely abolished. Interestingly, the corresponding single mutants contributed to this abolishment by approximately half each, suggesting these sites are independently involved in the regulation [14].

PKA has been shown to phosphorylate AQP1 in vitro in rat kidney homogenate, and studies in oocytes show that the cAMP and arginine vasopressin (AVP), both activators of PKA, increase AQP1 membrane abundance [45]. This would suggest that AQP1 phosphorylation could be regulated by AVP via cAMP-dependent PKA activation, as seen for AQP2 (see below). If AQP1 trafficking is controlled by PKA-phosphorylation, this would be additional to the main regulation by PKC as HEK293 cells expressing AQP1 (unlike collecting duct cells expressing AQP2) successfully translocate the protein into the plasma membrane, even though the cells lack the AVP receptor [99]. Furthermore, the hypotonicity-triggered trafficking of AQP1 in HEK293 cells was not impaired by a PKA-inhibitor [99], suggesting that the mechanisms of regulation by PKA and PKC are independent. More studies are needed to understand the role of PKA phosphorylation in AQP1 regulation, to identify the specific residue at which this phosphorylation occurs, and to understand the mechanism underlying trafficking for storage or degradation when an increased water/ion conductance is no longer required.

### 6.2. AQP2

**Figure 4:**
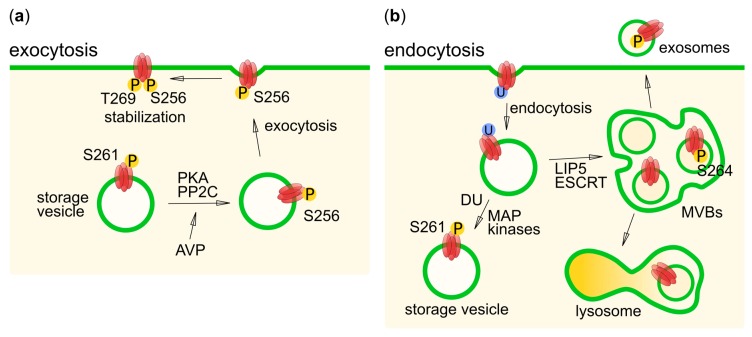
Schematic figure of the role of phosphorylation and ubiquitination in trafficking of AQP2 in the kidney collecting duct principle cell. AQP2 is represented in red, phosphorylation in yellow and ubiquitination in blue. DU stands for de-ubiquitination. (**a**) AQP2 exocytosis. Arginine vasopressin (AVP) stimulation results in activation of PKA and protein phosphatase 2C (PP2C), which phosphorylate S256, and dephosphorylate S261, respectively. The vesicle then fuses with the apical membrane resulting in an increase in urine concentration. The protein can be further stabilized in the plasma membrane by an additional phosphorylation of T269. (**b**) AQP2 endocytosis. Upon ubiquitination, AQP2 is internalized and may be recycled (when phosphorylated at S261) or targeted to multi-vesicular bodies (MVBs), with the help of LIP5 and the ESCRT machinery. Once in MVBs, fusion of the whole endosome with lysosomes result in AQP2 degradation. Alternatively, the MVB inner vesicles, containing AQP2 phosphorylated at S264, may be excreted into the urine as exosomes.

AQP2 is located in several tissues in the human body, but is best studied in terms of its function in the kidney collecting duct where it plays a key role in regulating urine volume. This relies on AVP-dependent trafficking of AQP2 to, and from, the apical membrane of the collecting duct principal cells, a mechanism that has been extensively studied and serves as the canonical example for hormone-induced regulatory exocytosis. During periods of dehydration, AVP is secreted from the pituitary gland and binds to the vasopressin 2 receptor in the basolateral membrane. This leads to an increase in intra-cellular cAMP and subsequent phosphorylation of the AQP2 by PKA. PKA-mediated phosphorylation triggers the translocation of AQP2 residing in storage vesicles to the apical membrane, causing increased water uptake from urine (Figure 4a) [47]. Once hydration levels have been restored, dephosphorylation and mono-ubiquitination cause AQP2 to be internalized, after which it may be stored again in storage vesicles, targeted to lysosomes for degradation or secreted as exosomes (Figure 4b) [100].

AVP-dependent trafficking of AQP2 relies on an intricate interplay between multiple phosphorylation sites in the AQP2 C-terminus: S256, S261, S264 and T269 (S269 in mouse), all of which have been shown to be phosphorylated in vivo (Figure 1c and Table 1) [49]. Of these, S256 plays a prominent role, as phosphorylation of this residue by PKA is necessary and sufficient for targeting AQP2 to the apical membrane [47]. Once in the apical membrane, AQP2 may be subsequently phosphorylated at T269, leading to an increase in AQP2 retention time [101], as shown by the decreased internalization rates of the double S256D/S269D mouse AQP2 mutant in Madine-Darby canine kidney (MDCK) cells [22]. The kinase responsible for T269 phosphorylation remains to be identified, but it has been speculated that, although it does not sit in a canonical PKA site (RXS/T^269^ instead of RRXS/T), it may nevertheless be phosphorylated by PKA [48,49]. 

The role of the two remaining phosphorylation sites, S261 and S264, has not been completely elucidated. In contrast to S256 and T269, AVP stimulates the dephosphorylation of S261 by protein phosphatase 2C [102], and AQP2 phosphorylated at this residue mainly resides in intra-cellular vesicles [49,103,104]. It was further shown that S261 phosphorylation occurs after ubiquitin-induced endocytosis and may stabilize ubiquitinated AQP2 [104]. The reduced phosphorylation of S261, following an AVP-signal has been proposed to be the result of a cAMP-dependent inhibition of p38-mitogen activator protein (MAP) kinase, due to its phosphorylation by PKA. The involvement of proline-dependent kinases, such as the MAP kinase family, is supported by the presence of a proline residue immediately following S261 and an AQP2 C-terminal peptide could be phosphorylated in vitro, by several MAP kinases, including p38 [51].

As for S256 and T269, AVP stimulates phosphorylation of S264, which is suggested to sit in a casein kinase type I phosphorylation site (SXXS where the first S is phosphorylated) or a PKC site [48,49]. S264 phosphorylation has been shown to be associated with localization in both the apical and basolateral membrane after short-term AVP-treatment (<15 min), whereas prolonged treatment (>60 min) has resulted in localization to the apical membrane and early endosomes. Since AQP2 phosphorylated on S264 was not seen in lysosomes, it was proposed that it may play a role in excreting AQP2 in exosomes. [105]. Interestingly, interaction studies between AQP2 and the MVB-sorting protein LIP5 showed that, in contrast to other phospho-mimicking mutants, which are destined for the apical membrane or recycling vesicles, AQP2 S264E had similar affinity to LIP5 as non-phosphorylated AQP2 [21]. This implies that AQP2 phosphorylated at S264, may be incorporated into MVB inner vesicles. Since exosomes are believed to originate from MVB inner vesicles, this supports a role for S264 phosphorylation in AQP2 exosome excretion.

A few studies have tried to elucidate the role of specific phosphatases in AQP2 trafficking. In PKA-phosphorylated endosomes isolated from rat kidney collecting duct the AQP2 could be dephosphorylated, in vitro, by the addition of protein phosphatase 2B (calcineurin) [60]. Calcineurin-null mice exhibit normal AQP2 expression, but decreased levels of AQP2 phosphorylation and increased retention in intra-cellular compartments in response to vasopressin. This led the authors to conclude that calcineurin is essential for normal trafficking of AQP2 [61]. In the presence of inhibitors of PP1 and PP2A phosphatases, the abundance of AQP2 phosphorylated at S256, S264 was increased in unstimulated rat inner medullas ex vivo. Furthermore, the abundance of phosphorylated S261, and S264 was increased, when calcineurin was inhibited [62]. Protein phosphatase 2C was found to be required for an effective dephosphorylation of S261 upon vasopressin stimulation, as described above [102].

### 6.3. AQP4

The effect of phosphorylation on AQP4 function was first suggested already 20 years ago [31] and has profound clinical impact due to its connection with development of brain edema [106]. Since then the immense complexity of this regulation has become evident, with 5 different kinases and different phosphorylation sites proposed to date: S111 and S180 in loop B and D respectively and S276, S285, S315, S316, S321 and S322 within the C-terminus, the latter which have all been shown to be phosphorylated in vivo (Figure 1c and Table 1) [13]. As described earlier in this review there is some evidence for phosphorylation-dependent gating of AQP4 involving S111 and S180. Here we describe the current evidence for phosphorylation-dependent trafficking of AQP4, focusing on the mostly studied sites S180 and S276.

Phosphorylation of S180 by PKC has been shown to lead to increased AQP4 internalization. Upon treatment with PKC activators, the water content of rat brain was significantly reduced ex vivo, an effect that correlated with AQP4 downregulation [88]. PKC activators also caused reduced cell swelling in oocytes [27,31], LLC-PK1 kidney cells [26], and human brain tumour cells [28], an effect that was partly abolished when S180 was mutated to alanine [26,27,28]. It has further been suggested that PKC-induced internalization in AQP4 depends on AVP, which upon binding to the vasopressin 1_a_ receptor (V1_a_R), activates a signalling cascade ending with PKC activation. Indeed, oocytes co-expressing both AQP4 and V1_a_R, were sensitive to AVP and upon AVP treatment, the water permeability was reduced two-fold. In addition to PKC, PKA has also been proposed to regulate AQP4 internalization. Gastric cells expressing rat AQP4 were treated with histamine, resulting in reversible internalization of AQP4 which co-localized with late endosomes but not with lysosomes [54]. While the initial internalization was PKA-independent, subsequent phosphorylation was dependent on PKA and independent of PKC and CKII, however the specific phosphorylation site could not be determined. Taken together, it is possible that phosphorylation by PKC marks the protein for internalization, while phosphorylation by PKA helps to retain the protein in internal vesicles [27].

Contradicting these results, studies in HEK293-cells did not show a difference in constitutive expression or hypotonicity-triggered translocation of AQP4 S180A or S180D, nor any effect of PKC-inhibitors. Instead, it was shown that AQP4 plasma membrane targeting was dependent on PKA, as hypotonicity-induced translocation of AQP4 to the plasma membrane was abolished by PKA inhibitors (but not by PKC inhibitors) in both HEK293 cells and primary astrocytes. The PKA-phosphorylation event was attributed to S276 in the C-terminus, since a S276A mutant did not translocate to the membrane following a hypotonic trigger. The corresponding phospho-mimicking mutant S276D could reach the membrane also in the presence of PKA inhibitors, suggesting that phosphorylation at other PKA-sites is not necessary for translocation to occur. Interestingly, directly upstream of this phosphorylation site a putative CaM-binding site was identified. CaM is known to modulate the water permeability of AQP4, however it is not known whether this happens through direct binding or indirectly through protein kinase activation [25].

Other studies have shown that phosphorylation of S276 has the opposite effect on AQP4 trafficking. This residue has also been identified as a casein kinase II consensus site and a S276D mutant has been shown to have 5-fold increased affinity to the μ3A subunit of the AP3 complex in a yeast two-hybrid assay, with S276 immediately preceding the tyrosine-based binding site. This interaction plays an important role in targeting proteins for lysosomal degradation following clathrin-mediated endocytosis. Moreover, the same mutant showed an increased co-localization with lysosomes in MDCK cells. Neither the delivery rate to the plasma membrane nor protein internalization rate was affected by this phosphorylation, suggesting it plays a role in intracellular sorting of AQP4 [37].

In summary, studies of phosphorylation-dependent trafficking of AQP4 have generated conflicting results and more work will be needed to dissect the roles of individual kinases and phosphorylation sites. It seems clear that there are multiple pathways that regulate AQP4 membrane abundance and that the existence of these pathways, and/or the cross-talk between them, may differ depending on the experimental conditions or the cell type used.

### 6.4. AQP5

AQP5 is highly expressed in salivary, sweat, lacrimal glands, as well as lungs and airways and plays a key role in secretion of isotonic fluids [107]. Similarly as for its closest homologue AQP2, AQP5 has been shown to be regulated by trafficking in a cAMP- and PKA-dependent manner in several cell types [19,23,55,108,109,110], and PKG activity has been suggested to regulate the acetylcholine-triggered trafficking of AQP5 [57], although further details on the latter are currently lacking. Exposure to cAMP has a bi-phasic effect on AQP5 translocation: Increased internalization and decreased total abundance in the short-term (minutes), and increased plasma membrane targeting in the long-term (hours), both of which are dependent on PKA, but where only the long-term effect involves direct AQP5 phosphorylation [111]. Two consensus PKA sites are found in AQP5, S156 in loop D and T259 in the C-terminus (Figure 1c), the latter which correspond to S256 in AQP2 (Figure 3), where it plays a crucial role in AVP-mediated membrane targeting (see above). Both these sites have been shown to be able to be directly phosphorylated by PKA and S156, was shown to be preferentially phosphorylated in tumor cells [112].

Several studies have probed the individual roles of S156 and T259 in PKA-dependent AQP5 trafficking. In MDCK-II cells, S156 as well as the immediately preceding consensus phosphorylation site S152 was mutated to alanines, with the mutant exhibiting only slightly increased trafficking to the plasma membrane [23]. This was further supported by studies in HEK293-cells, where a S156A mutant did not show altered membrane expression compared to wild-type AQP5. However, in the same study, the corresponding S156E phospho-mimicking mutant had an increased membrane localization, showing that phosphorylation of S156 does play a role in AQP5 membrane targeting. Since S156A behaved as wild-type, this indicates that AQP5 may not be phosphorylated at S156 under basal conditions. Treatment with a PKA-inhibitor increased the AQP5 plasma membrane abundance of wild-type AQP5 as well as both S156A and S156E mutants, suggesting an additional PKA-dependent regulation step, independent from S156 phosphorylation. This second event may therefore involve phosphorylation of a different target than AQP5 or the second PKA-site, T269 [19]. The latter is however contradicted by studies in MDCK-cells and mouse salivary gland cells, where phosphorylation of T259 was not important for AQP5 exocytosis following cAMP stimulation, as shown by wild-type AQP5, and an T259A mutant exhibiting the same translocation behaviour [34,56].

Taken together, these studies support the biphasic response on AQP5 trafficking following cAMP-stimulation, with the short-term effect causing a decrease in AQP5 membrane abundance through phosphorylation of a different target and the long-term effect resulting in increased AQP5 membrane levels, involving direct phosphorylation of S156. Structural studies of the AQP5 S156E-mutant showed that, contradictory to what was originally proposed from the crystal structure of wild-type AQP5 [113], phosphorylation of this residue does not cause any structural changes to the protein [19]. It is therefore likely that it is the presence of the phosphate group itself rather than a conformational change that is recognized as a sorting signal. Interestingly, AQP5 was also shown to rapidly translocate to the membrane in response to a hypotonic trigger in a manner that was independent of PKA and S156 phosphorylation [19]. Whether this involves phosphorylation by PKC, as shown for hypotonicity-induced translocation of AQP1 [24], remains to be shown.

## 7. Concluding Remarks

The AQPs constitute one of the best characterized families of integral membrane proteins in terms of the structure. While this has led to a thorough understanding of the structural basis for transport and selectivity, much remains to be learnt about how different AQPs can be specifically regulated, despite sharing a conserved structural fold. The regions important for AQP regulation are most often found outside the highly conserved transmembrane core domains, in the loops and termini. These regions are often highly flexible, and the structural details and their involvement in regulation may be difficult to deduce from a static structure. In fact they are often missing from AQP structures, due to disorder.

Located within the loops and termini, phosphorylation sites play crucial roles in the differential regulation of human AQP isoforms. Through phosphorylation/dephosphorylation, the structures and properties of these regions are altered, changing their ability to bind the regulatory proteins that control the phosphorylation-dependent regulatory events, including translocation to and from the plasma membrane, targeting degradation and, in the case of gating, whether the AQP is open and closed. It seems likely that the flexibility is one of the key properties that allow these regions to play such prominent roles in AQP regulation and they may often lack a stable secondary structure in the absence of an interacting partner. In this respect, obtaining structural information for AQPs in complexes with regulatory proteins will be crucial in order to understand the roles of these regions and specific phosphorylation sites in AQP regulation.

An additional layer of complexity in AQP regulation becomes apparent when considering an even higher level of regulation: The regulation of kinases and phosphatases themselves. Much remains to be determined, regarding which specific enzymes are involved and how they are regulated. Such information would significantly aid in our understanding of the cross-talk between different kinases and phosphatases when regulating AQPs with multiple phosphorylation sites, or when several AQPs are present and differentially regulated within the same cell.

## Figures and Tables

**Table 1:** Kinases and phosphorylation sites involved in regulation of mammalian AQP0-9. PKA—protein kinase A; PKC—protein kinase C; PKG—protein kinase G; CK—casein kinases; CaMKII—calmodulin-kinase II; N/A—residue information not available. Phosphorylation of AQP6 has to our knowledge not been identified.

AQP	PKA	PKC	PKG	CK	CaMKII	Other	Unknown
AQP0	S235 [42]	S231 [43] S235 [44]		S229 [43]			
AQP1	N/A [45]	T157 [14,24] T239 [14,24]					Y253 [46]
AQP2	S256 [47] T269 ^1^ [48]	S264 ^1^ [49]	S256 [50]	S264 ^1^ [48]		S261—MAP kinases [51]	
AQP3		Indirect [52]					
AQP4	Indirect [53] N/A [54] S276 [25]	S180 [26,27]	S111 [32]	S276 [37]	Indirect [32,41]		S285, S315, S316, S321, S322 [13]
AQP5	S156 [55] T259 [56]		N/A [57]				
AQP6							
AQP7	S10 [15] T11 [15]						
AQP8	N/A ^2^ [58]	N/A ^2^ [59]					
AQP9		S11 [40]					

^1^ Remains to be conclusively established; ^2^ Possibly an indirect effect.
